# New scenarios in secondary hyperparathyroidism: etelcalcetide. Position paper of working group on CKD-MBD of the Italian Society of Nephrology

**DOI:** 10.1007/s40620-019-00677-0

**Published:** 2019-12-18

**Authors:** Antonio Bellasi, Mario Cozzolino, Fabio Malberti, Giovanni Cancarini, Ciro Esposito, Carlo Maria Guastoni, Patrizia Ondei, Giuseppe Pontoriero, Ugo Teatini, Giuseppe Vezzoli, Marzia Pasquali, Piergiorgio Messa, Francesco Locatelli

**Affiliations:** 1 UOC Ricerca, Innovazione, Brand Reputation, ASST-Papa Giovanni XXIII, Bergamo, Italy; 2 UOC Nefrologia e Dialisi ASST Santi Paolo e Carlo, Department of Health Sciences, University of Milan, Milan, Italy; 3 Struttura Complessa di Nefrologia e Dialisi, Istituti Ospedalieri di Cremona, Cremona, Italy; 4 U.O.C. Nefrologia e Dipartimento della Cronicità, ASST, Spedali Civili e, Università di Brescia, Brescia, Italy; 5 Struttura Complessa di Nefrologia e Dialisi, ICS Maugeri SpA SB, Università di Pavia, Pavia, Italy; 6 UO Nefrologia ASST Ovest Milanese, Milan, Italy; 7 USS Emodialisi, Azienda Ospedaliera Ospedale Papa Giovanni XXIII, Bergamo, Italy; 8 Ospedale “Alessandro Manzoni”, ASST Lecco, Lecco, Italy; 9 UOC Nefrologia e Dialisi. ASST Rhodense, Garbagnate M.se, Italy; 10 Unità di Nefrologia e Dialisi, IRCCS Istituto Scientifico San Raffaele, Università Vita Salute San Raffaele, Milan, Italy; 11 UOC di Nefrologia-Azienda Ospedaliero-Universitaria Policlinico Umberto I Roma, Rome, Italy; 12 Unità Operativa Complessa di Nefrologia e Dialisi, Fondazione IRCCS Ca’ Granda Ospedale Maggiore Policlinico, Milan, Italy

**Keywords:** Secondary hyperparathyroidism, Etelcalcetide, Cinacalcet, CKD-MBD, PTH

## Abstract

Bone mineral abnormalities (defined as Chronic Kidney Disease Mineral Bone Disorder; CKD-MBD) are prevalent and associated with a substantial risk burden and poor prognosis in CKD population. Several lines of evidence support the notion that a large proportion of patients receiving maintenance dialysis experience a suboptimal biochemical control of CKD-MBD. Although no study has ever demonstrated conclusively that CKD-MBD control is associated with improved survival, an expanding therapeutic armamentarium is available to correct bone mineral abnormalities. In this position paper of Lombardy Nephrologists, a summary of the state of art of CKD-MBD as well as a summary of the unmet clinical needs will be provided. Furthermore, this position paper will focus on the potential and drawbacks of a new injectable calcimimetic, etelcalcetide, a drug available in Italy since few months ago.

## Introduction

Secondary hyperparathyroidism (SHPT) is a common, severe and costly complication of Chronic Kidney Disease (CKD), and it has an unfavorable impact on outcomes of patients, particularly in those undergoing hemodialysis [1–3] (please refer to Table 1 for a complete list of abbreviations and acronyms).

**Table 1:** List of abbreviations and acronyms used in the present paper and relevant explanations

Abbreviation	Meaning
CAC	Coronary artery calcification
CaSR	Calcium sensing receptor
CKD	Chronic kidney disease
CKD-G5D	Chronic kidney disease under dialysis treatment
CKD-MBD	Chronic kidney disease mineral bone disorder
COSMOS	Observational clinical study in patients undergoing maintenance dialysis treatment in Europe
DOPPS	Dialysis examinations and practice patterns study
EAP	Efficacy assessment period
EVOLVE	Evaluation of Cinacalcet HCl therapy to lower cardiovascular events trial
FGF23	Fibroblast growth factor 23
iPTH	Intact Parathyroid hormone
KDIGO	Kidney disease improving global outcomes (the Organization that issued the guidelines)
PTH	Parathyroid hormone
SAPC	Serum albumin peptide conjugate
SHPT	Secondary hyperparathyroidism
VDR	Vitamin D receptor

Although many treatment approaches are available, remarkable proportions of patients still present inappropriate serum levels of parathyroid hormone (PTH), phosphate and calcium, often reaching far beyond what is recommended by the guidelines for the treatment of Chronic Kidney Disease Mineral Bone Disorder (CKD-MBD) [1, 4–6]. In the COSMOS study [4], a review of approximately 4500 subjects in 227 European dialysis centers, phosphate levels were higher than normal in 70% of dialysis patients, reaching values beyond 5.5 mg/dl in 41% of them. Similarly, only about one patient out of two (55.4%) in Italy present values of CKD-MBD parameters within the desired ranges [4]. In the US, based on the data from the Dialysis Outcomes Practice Patterns Study (DOPPS), a survey performed in 2015, phosphate levels were higher than recommended by the guidelines for CKD-MBD management in more than 60% of prevalent patients undergoing hemodialysis treatment longer than 180 days [6]. With regard to the plasma levels of PTH, according to DOPPS data, a progressive increase of median levels has been observed in the last years both in Europe and the US [5]. In the 2012–2014 timeframe of the survey, in particular, more than 25% of patients treated in the US presented with poorly controlled SHPT (defined as PTH level > 500 pg/ml) [5]. In line with these observations, hyperparathyroidism was reported in a recently published study in as much as 29% of patients receiving chronic hemodialysis in Italy [7]. In addition to such an assessment of the grade of appropriateness in the management of SHPT both in Italy and internationally, DOPPS data show that, in dialysis patients, there’s an increase in the risk of cardiovascular mortality and all-cause mortality associated with serum calcium levels > 10 mg/dl, phosphate levels > 7 mg/dl, and PTH levels > 600 pg/ml [8], so as to highlight how much an appropriate control of SHPT and CKD-MBD is still an unmet clinical need.

Current strategies for the medical treatment of SHPT are grounded in the following criteria: lower the intake of phosphate by careful avoiding food and beverages with “masked” phosphorus content (many preserved foods and alcohol-free beverages) [9, 10]; privilege the intake of proteins of vegetal origin which, due to higher content in fiber, cause lower intake of phosphorus so as to reduce animal proteins while avoiding malnutrition; use phosphate binders [10, 11]; use vitamin D analogs [7, 12, 13]; ensuring dialysis appropriateness in dialysis patients, with particular regard to duration; use of calcimimetics (Table 2). The latter are chemical agents which activate calcium sensing receptor (CaSR) and suppress PTH secretion from the parathyroid glands [1–3]. Parathyroidectomy is usually the last resort treatment and is only performed after evidence of failure of drug therapy [1–3, 7, 14], and a careful evaluation of all potential complications of such surgery procedure [15].

**Table 2:** Available marketed treatments for secondary hyperparathyroidism

Treatment	Formulation	Starting dose	Most common side effects
**Phosphate binders**			
Calcium based			
Calcium acetate^a^	667 mg capsules	667–1334 mg	Nausea, vomiting, elevated serum calcium
Calcium carbonate	250–1000 mg tablets	500–1000 mg	Nausea, vomiting, elevated serum calcium, diarrhea, abdominal pain, flatulence, constipation
Calcium acetate + magnesium carbonate	435 mg + 235 mg 180 film-coated tablets	3 to 10 film-coated tablets daily, depending on serum phosphate level	Nausea, loss of appetite, feeling of fullness, belching, constipation and diarrhea, loose stool, symptomatic or asymptomatic elevation of serum calcium, asymptomatic elevation of serum magnesium
Non calcium based			
Sevelamer HCl/carbonate	800 mg tablets, 2400 mg sachets (oral suspension powder)	800-1600 mg 3 times daily with meals	Headache, diarrhea, dyspeptic disorders
Lanthanum carbonate	250, 500, 750, 1000 mg chewable tablets	1500 mg daily	Diarrhea, nausea, abdominal pain, vomiting
Sucroferric oxyhydroxide	500 mg chewable tablets	1500 mg of iron (3 tablets) daily	Diarrhea, stool color change, nausea, vomiting, constipation
**Active vitamin D analogs**			
Calcitriol	0.25 mcg capsules	0.25 mcg daily	Elevated serum calcium, headache, abdominal pain, nausea, skin rash, urinary tract infections
	1 mcg injectable solution	1.0 mcg (0.02 mcg/kg) to 2.0 mcg 3 times weekly (every other day)	
Paricalcitol	1, 2, 4 mcg capsules	1-2 mcg (PTH < 500 pg/ml) or 2-4 mcg (PTH > 500 pg/ml) every other day	Diarrhea, arterial hypertension, dizziness, vomiting and elevated serum calcium
	5 mg/ml vials		
**Calcimimetics**			
Cinacalcet	30, 60, 90 mg tablets	30 mg daily	Reduced serum calcium, muscle spasms, diarrhea, nausea, vomiting
Etelcalcetide	2.5 mg, 5 mg, 10 mg vials	5 mg bolus injection 3 times weekly	Reduced serum calcium, muscle spasms, diarrhea, nausea, vomiting

^a^Available for patients on renal replacement therapy only

The goal of the treatment is to maintain calcium, phosphate and serum PTH below the levels advised by the guidelines for CKD-MBD management [2]. However, partly because of the limitations of the current therapeutic options for SHPT, a large proportion of patients on chronic replacement therapy do not reach target levels of PTH [4–6]. It is not thus surprising that the research in this field is still ongoing with the aim of developing molecules able to offer new therapeutic solutions for clinicians to cure bone mineral disorders [3].

In this “Position Paper”, we are going to present the updates of the guidelines for CKD-MBD management issued by Kidney Disease Improving Global Outcomes (KDIGO) and the evidence available on etelcalcetide, a novel calcimimetic agent administered intravenously, which a few months ago entered the therapeutic armamentarium of nephrologists who are struggling with the management of SHPT.

### KDIGO guidelines on CKD-MBD management: new updates

The updates of the guidelines for diagnosis, evaluation, prevention and treatment of CKD-MBD in patients with chronic kidney disease were released in August 2017 [16] at the end of a long internal review process. In fact, the changes from previous 2009 guidelines are modest, particularly with regard to management and therapy of SHPT [16].

Among the new recommendations, the importance of serial assessments of phosphate, calcium, and PTH levels considered together, is reiterated under point 4.1.1 of the guidelines [16]. When deciding or modifying the treatment of CKD-MBD, it is indeed important to consider the set of these biochemical markers as a whole, and to also take into account their trends in time, an aspect that is often neglected by clinicians. In patients in dialysis (CKD-G5D) it is suggested to maintain phosphate levels in the normal range, avoid hypercalcemia, and use a dialysate calcium concentration between 1.25 and 1.50 mmol/l (grade and strength of the recommendations: 2C) [16]. Concerning management and adjustment of PTH levels, the recommendation (under point 4.2.3, grade and strength 2C) is unchanged from year 2009 [2, 16], i.e. it is suggested to maintain PTH levels in the range of 2–9 times the upper normal limit reported by the lab and, importantly, to initiate or change therapy in case of progressive changes in PTH levels, even if still within the suggested range. This recommendation applies for either increased or decreased PTH levels, in order to avoid both hypercorrection and suboptimal treatment of SHPT [16]. As it is shown in Table 2, many drugs are available to improve SHPT, and guidelines suggest to use calcimimetics, calcitriol, vitamin D analogs or a combination of these in patients in dialysis (point 4.2.4, 2B) [16]. The choice of the treatment and the assessment of the best therapeutic strategy are up to the physician in charge of the single patient, based on his/her biochemical pattern, as it is reported under the above mentioned point 4.1.1 of the guidelines [2, 16].

Among the working group who reviewed the KDIGO 2017 guidelines there was no consensus as to the indication for the use of calcimimetics as first line treatment in CKD-G5D and SHPT patients (Table 3). Several studies show superior efficacy of cinacalcet as compared to active vitamin D or its analogs for the simultaneous control of both PTH and the other bone mineral metabolism markers (serum calcium, serum phosphate, FGF23) [17–23]. Therefore, in our opinion calcimimetics should be seen as first line drugs to control SHPT in patients with moderately to severely elevated serum calcium levels and elevated levels of phosphate, while active vitamin D or its analogs should be used as first choice in patients with hypocalcemia. However, it is necessary to remind that in most cases the best control of SHPT is achieved in the later phase using a balanced combination of calcimimetics and low doses of active vitamin D or its analogs.

**Table 3:** Main messages arising from the latest KDIGO review of the guidelines on CKD-MBD management [16, 17]

KDIGO guidelines
It is important to evaluate the set of biochemical markers as a whole and their trends in time
Hypercalcemia should be avoided
Calcimimetics are among first choice options to consider in stage-5D CKD patients

Indeed, several randomized clinical trials have proven that combined therapy with cinacalcet and low doses of active vitamin D might achieve better control of SHPT in comparison with solely using active analogs of vitamin D [17–23].

In a study by Urena et al. [18] conducted in 300 incident patients undergoing hemodialysis, combined therapy with cinacalcet and low doses of active vitamin D (average weekly dose of paricalcitol 4.5 mcg) achieved greater reduction of PTH levels, along with lower average levels of serum calcium and phosphate (8.7 vs. 9.3 mg/dl and 5.1 vs. 5.5 mg/dl respectively) as compared with monotherapy with paricalcitol (average weekly dose 11.7 mcg).

In the IMPACT study [23], the control of PTH levels was similar between subjects treated with cinacalcet and low doses of vitamin D and those who were treated with monotherapy either with paricalcitol or analogs of vitamin D. However, in comparing study-end and baseline serum levels, a trend was observed toward a decrease in phosphate, calcium and FGF23 among patients treated with cinacalcet, while the same markers were increased by the use of intravenous or oral paricalcitol. Furthermore, in around 11% of patients treated with paricalcitol, hypercalcemia (defined as serum calcium over 11 mg/dl) was detected.

Also the PARADIGM study [20], conducted in 312 dialysis patients, showed comparable control of PTH levels between the group treated with cinacalcet (median daily dose at 52 weeks 85 mg) and the one treated with paricalcitol (median weekly dose at 52 weeks 21 mcg). However, serum calcium, phosphate and FGF23 were significantly reduced in patients treated with cinacalcet, while the same markers were increased in the paricalcitol group [20].

According to the “Evidence based medicine”, the EVOLVE study (Evaluation of Cinacalcet HCl Therapy to Lower Cardiovascular Events) [17], the largest placebo-controlled double-blind clinical trial ever conducted in dialysis patients with SHPT, was not able to prove superiority of cinacalcet over placebo in achieving the more ambitious composite primary end-point of all-cause mortality, myocardial infarction, unstable angina requiring hospitalization, heart failure or peripheral vascular disease [17]. The statistical inconclusive significance of the study is due to the reduction of its statistical power from 90% to 54% [24], as a consequence of both the decision of the physicians in charge to treat placebo patients with a calcimimetic, and the side-effects drop-out rate from the calcimimetic drug, mainly due to gastrointestinal events.

Therefore, on the one hand clinical trials using surrogate endpoints like biochemical markers control have proven a specific pattern achieved by the calcimimetic; on the other, the EVOLVE study [17] could not prove an impact of CaSR modulation on hard endpoints. In fact, at the end of the 64-month treatment period, the reduction of the composite risk of all-cause mortality, myocardial infarction, unstable angina requiring hospitalization, heart failure or peripheral vascular disease was proven non-significant (Hazard Ratio 0.93; 95% Confidence Interval 0.85–1.02; p = 0.11) [17]. Nevertheless, in the EVOLVE study the risk of developing severe and unremitting hyperparathyroidism and undergo parathyroidectomy was shown to be lower in the cinacalcet group than in controls (who were treated with active vitamin D and/or phosphate binders) [25]. This observation, however, is based on secondary or post hoc analysis and it requires further evidence. Other interesting findings from the EVOLVE study are listed in Table 4 [26–30]. Treatment with cinacalcet was more beneficial in individuals of age over 65 years as compared to the younger ones [29]. In general, the risk of calciphylaxis [30], non-atherosclerotic cardiovascular events [27], calcifications [28], and bone fractures [26] seemed to be lower when CKD-MBD therapy included the use of a CaSR modulator.

**Table 4:** Key findings from the evalution of Cinacalcet HCL Therapy to Lower Cardiovascular Events (EVOLVE) Trial

Primary endpoint	
Composite risk of all-cause mortality, myocardial infarction, unstable angina requiring hospitalization, heart failure or peripheral vascular disease (not adjusted for confounding factors)	HR 0.93; (95% CI 0.85–1.02) [11]
Secondary endpoints and post hoc analyses	
Composite risk of all-cause mortality, myocardial infarction, unstable angina requiring hospitalization, heart failure or peripheral vascular disease (adjusted for confounding factors)	**HR 0.88; (95% CI 0.79–0.98) [**11**]**
Composite risk of all-cause mortality, myocardial infarction, unstable angina requiring hospitalization, heart failure or peripheral vascular disease (lag censoring)^a^	**HR 0.85; (95% CI 0.76–0.95) [**11**]**
Composite risk of all-cause mortality, myocardial infarction, unstable angina requiring hospitalization, heart failure or peripheral vascular disease in individuals older than 65 years of age	**HR 0.70; (95% CI 0.60–0.81) [**22**]**
Composite risk of all-cause mortality, myocardial infarction, unstable angina requiring hospitalization, heart failure or peripheral vascular disease in individuals younger than 65 years of age	HR 0.97; (95% CI 0.86–1.09) [22]
Risk of bone fractures, unadjusted	HR 0.89; (95% CI 0.75–1.07) [19]
Risk of bone fractures, adjusted for potential confounding factors	HR 0.83; (95% CI 0.72–0.98) [13]
Fatal and non-fatal non-atherosclerotic/ischemic cardiovascular events (e.g. arrhythmia)	**HR 0.84; (95% CI 0.74–0.96) [**20**]**
Risk of parathyroidectomy or severe SHPT (unremitting)	**HR 0.31; (95% CI 0.26–0.37) [**18**]**
Risk of calciphylaxis	HR 0.31; (95% CI 0.13–0.79) [23]

Summarized results include Hazard Ratios (HR) for the comparison between cinacalcet and placebo

Statistically significant results are in bold

^a^For a full presentation of the results of the EVOLVE trial please refer to Locatelli F, et al. What can we learn from a statistically inconclusive trial? Consensus conference on the EVOLVE study results. G Ital Nefrol 2013 Sep-Oct;30 [5]. pii: gin/30.5.4 [31]

In summary, the current clinical evidence does not allow to draw clear indications on which therapy of SHPT should be chosen as first line, but it actually suggests that the treatment strategy should be tailored to the biochemical pattern of each patient, leaving physicians free to make the clinical choices they deem appropriate for their patients. Clinical common sense tells us we should follow the suggestions here above.

### When and how to start drug treatment of SHPT?

The KDIGO guidelines include only very general advices about when to initiate treatment with drugs able to reduce the levels of PTH. At point 4.2.3, among other things, the KDIGO guidelines [2, 16] suggest to start treatment when repeated increases of PTH levels are observed, even within the suggested range, in order to avoid progression of the condition of SHPT [2, 16].

In clinical practice, however, such suggestion does not seem to be much followed [5]. The latest DOPPS data show that SHPT treatment is generally started late, namely, when PTH has exceeded the recommended target [5, 6]. In contrast with the increase of the average levels of PTH observed during the study period, prescription of active vitamin D was frequent (around 80% of patients were treated with vitamin D) but it remained stable. Changes were not seen either in the prescription of cinacalcet, that remained stable and limited to around 20% of white and 37% of black patients [5, 6]. Although cinacalcet use has been associated with parathyroid gland volume reduction even in patients with marked parathyroid hyperplasia [31], there are physiopathological and clinical considerations that should push to start treating SHPT as early as possible, with the aim of achieving optimal and simultaneous control of multiple mineral metabolism markers and slow and/or contain the progression of SHPT [1, 3]. In detail, it is thought that chronic and abnormal stimulation of the parathyroid glands to release PTH might lead to structural changes of the glands themselves which, with time, become progressively less sensitive to mechanisms regulating PTH production and therefore also to medical therapy (unremitting SHPT) [32, 33]. Delaying treatment of SHPT might therefore mean to jeopardize its efficacy [32, 33].

There are substantial differences, though, among classes of drugs able to control PTH. When deciding the treatment for CKD-MBD, it is important to take these differences into account. In particular, the effect of the administration of vitamin D on calcium and phosphate balance can be very different from that of a CaSR modulator [34–41]. We know that, in dialysis patients, serum levels of calcium and phosphate do not only depend on dietary intake and intestinal absorption but also on dialysis balance and bone resorption of these elements [40, 41]. Protein intake being equal, for example, the risk of hyperphosphatemia increases with PTH levels [42–44]. In detail, in patients with protein intake from 0.8 to 1.2 g/kg/day the risk of hyperphosphatemia increases significantly when levels of PTH are > 300 pg/ml (as compared to the population with PTH levels from 150 to 300 pg/ml) [43].

In another study conducted in more than 100,000 patients undergoing maintenance dialysis treatment, the risk of hyperphosphatemia (defined as serum phosphate levels > 5.5 mg/dl), hypercalcemia (defined as serum calcium levels > 10.2 mg/dl) and elevated alkaline phosphatase (> 120 U/L) increased progressively with PTH levels [44], thus showing an important role of bone turnover in determining serum levels of these elements [44].

As compared to active analogs of vitamin D, cinacalcet does not increase the intestinal absorption of phosphate and is effective in reducing elevated bone turnover [34–41]. The action on bone turnover might indeed explain the finding of serum levels of calcium and phosphate generally lower in patients treated with a CaSR modulator than in those treated with vitamin D or its analogs [34–41].

Based on the available evidence and the physiopathological arguments discussed above, it seems reasonable to initiate treatment before the condition of unremitting SHPT is reached, using one or more drugs enabling the control of multiple biochemical markers at the same time.

### Calcimimetics: when and how to use these molecules? Suggestions from the working group

As it was discussed above, calcimimetics are a first line therapeutic option for the treatment of SHPT in patients with moderately to severely elevated serum calcium levels and elevated levels of phosphate, who are receiving renal replacement therapy [2, 39]. Drugs belonging to this class bind and allosterically activate CaSR (allosteric agonists) [2, 39]. So far cinacalcet has been the only drug in this class available for therapy. Since few months ago also etelcalcetide is available in Italy; as compared to cinacalcet, the new drug has a different administration route (intravenous) and a different mechanism of CaSR activation. Furthermore, other molecules are under clinical development or are ready to enter the market [2, 39].

Unlike vitamin D and its analogs, calcimimetics are effective in reducing PTH without increasing the intestinal absorption of calcium and phosphate [34–41]. Clinical studies have shown that treatment of SHPT with cinacalcet significantly reduces circulating levels of PTH [45]. After 26 weeks of treatment with cinacalcet or placebo, levels of iPTH ≤ 250 pg/ml were achieved in a remarkable proportion of patients treated with the drug (43%) as compared to placebo (5%) [45], and also better control of serum calcium and phosphate, in association with higher suppression of PTH, were observed [45]. Further studies have confirmed the efficacy of PTH suppression with cinacalcet, in combination with vitamin D or vitamin D analogs, as compared to monotherapy with calcitriol [46].

In the ACHIEVE study, PTH levels were reduced by at least 30% from baseline in 68% of patients receiving cinacalcet and only in 36% of patients receiving active vitamin D as monotherapy [47]. In another open-label 16-week study, 71% of patients treated with active vitamin D in combination with a low dose of cinacalcet reached levels of PTH ≤ 300 pg/ml as compared to 22% of patients treated with vitamin D only [48].

In all these studies mild to moderate hypocalcemia was common but easily manageable [49]. Hypocalcemia is a side effect attributed to CaSR modulation and likely to a reduction of bone calcium mobilization due to stricter control of PTH [49].

Lastly, the EVOLVE study has confirmed that achieving target levels is easier in CKD-MBD patients who are treated with cinacalcet than in the ones treated with placebo [17, 25]. As we already discussed, despite the primary endpoint of the study (all-cause mortality, myocardial infarction, unstable angina requiring hospitalization, heart failure or peripheral vascular disease) was not reached, serum levels of iPTH, calcium, phosphate and FGF-23 were better controlled among patients assigned to the cinacalcet treatment group [19, 25]. As compared to the placebo group, the most common side effects related to treatment with calcimimetics were nausea, vomiting and hypocalcemia; the prevalence of hypocalcemia was 12% in the cinacalcet group and 2% in the placebo group [17]. These side effects likely contributed to the higher discontinuation rate due to adverse events (16% in the cinacalcet group versus 12% in the placebo group) [24].

Activation of CaSR can have beneficial effects through the control of serum biochemical markers [1, 22, 24, 36, 39, 50]. Although inconclusive, the ADVANCE study, a phase 4 randomized clinical trial [22], showed a trend towards slower progression of cardiovascular calcifications in subjects treated with cinacalcet and low doses of active vitamin D, as compared to subjects treated with varying doses of vitamin D as monotherapy [22]. Following analyses showed that patients who were adherent to therapy or presented with large coronary and/or heart valve calcifications, seemed to benefit more greatly from this combined therapy [18]. In a post hoc analysis, subjects with aortic valve calcification treated with CaSR modulators showed a 74% reduction in the risk of progression of CAC (Odds Ratio 0.26; 95% CI 0.10–0.64) [51]. Also in the ADVANCE study, the most common side effects reported by subjects assigned to treatment with cinacalcet were gastrointestinal disorders and hypocalcemia, which occurred in 21% and 7%, respectively, of patients treated with this drug [22].

Although the activation of CaSR is an effective therapeutic strategy to control SHPT, further studies are needed to clarify the effect of potential interactions with various drugs commonly used to control CKD-MBD. According to a recent post hoc analysis of the INDEPENDENT study data [35], the concomitant administration of cinacalcet and Sevelamer significantly modulated the impact of these drugs on mortality (p = 0.006): treatment with cinacalcet, with or without Sevelamer, yielded longer survival (Hazard Ratio 0.34, 95% CI 0.14–0.81, p = 0.01) than the combined treatment of cinacalcet and calcium-based phosphate binders (Hazard Ratio 1.28, 95% CI 0.82–2.00, p = 0.26) [35]. Although further verification is required, these data suggest that the activation of CaSR might modulate balance of calcium [34] and affect survival of patients with CKD. Overall, however, the scientific evidence accumulated so far points to the need of a careful selection of the appropriate therapeutic strategy in SHPT.

### CKD-MBD, a field of intense research: new scenarios

On the date of January 26th 2018, 160 trials in SHPT were reported in the clinical trials register (www.clinicaltrials.gov; keyword: secondary hyperparathyroidism); 143 were completed or early terminated, and 17 were actively ongoing. Of these, 16 were interventional studies (four in phase 1, one in phase 2, three in phase 3, three in phase 4, and five unclassified), and one was listed as an observational study. Such numbers show that treatments and interventions targeting the mechanisms of development and progression of SHPT are a current area of interest.

Etelcalcetide (formerly denominated AMG 416) is a new prolonged-action drug, whose molecule is composed by a linear chain of seven amino acids, with the ability to activate CaSR (calcimimetic) [52–55]. Etelcalcetide binds directly to CaSR, inhibiting the production and secretion of PTH by parathyroid glands [52–55]. Such action is due to the formation of a disulfide bridge between d-cysteine in the etelcalcetide molecule and l-cysteine in CaSR, resulting in a fast activation of receptors [52–55]. Many scientific articles have been published in the last 2 years concerning pharmacokinetics, biotransformation and excretion of the drug both in animal models and in CKD patients undergoing hemodialysis treatment [52–55]. A summary of the main characteristics of etelcalcetide compared with cinacalcet is reported in Table 5.

**Table 5:** Summary of the differences in clinical and pharmacological properties between etelcalcetide (AMG 416) and cinacalcet

Etelcalcetide	Cinacalcet
Pharmacokinetics	
Composed by 8 synthetic amino acids (molecular weight 1048 g/mol)	Small organic molecule (molecular weight 393 g/mol)
Interacts with the extracellular domain of CaSR and reduces PTH secretion	Interacts with the intramembrane domain of CaSR and reduces PTH secretion
Long term action	Short term action
Clinical use	
Intravenous administration	Oral administration
Three times weekly at the end of dialysis	Daily use
Better compliance	Worse compliance
Greater PTH suppression	Lower PTH suppression^a^
Higher incidence of asymptomatic hypocalcemia	Lower incidence of asymptomatic hypocalcemia
Similar incidence of gastrointestinal effects (nausea, vomiting, diarrhea)	Similar incidence of gastrointestinal effects (nausea, vomiting, diarrhea)

^a^In the study comparing the drugs, 52.4% of patients treated with etelcalcetide showed a reduction of at least 50% in PTH levels at the end of a 26-week period of treatment vs. 40.2% of patients treated with cinacalcet (difference 10.2%, p = 0.001) [58]

Biotransformation of circulating etelcalcetide mainly yields a covalent conjugate with serum albumin (SAPC) [52–56]. The process of biotransformation in the human bloodstream is reversible, but the rate of formation of SAPC from etelcalcetide is faster than the inverse process [52–56]. These properties account for reduced extrarenal elimination and longer blood half-life of the drug [52–56]. When etelcalcetide was intravenously administered to CKD patients under chronic replacement therapy, the drug predominant clearance route was the dialysis treatment itself. Around 60% of the dosage was indeed found in the dialysate [52–56]. Based on these considerations, etelcalcetide should be given after the dialysis session in order to avoid the elimination of a substantial fraction of the administered dose and achieve long duration of action of the drug [52–56]. Similarly, due to elimination of a substantial fraction of the administered etelcalcetide dose in the presence of a significant residual renal function, together with the route (intravenous) and frequency (three times per week) of administration make this drug not suitable for treating hyperparathyroidism in peritoneal dialysis patients.

The efficacy of etelcalcetide was tested in several clinical trials versus placebo or cinacalcet in patients with CKD-G5D and SHPT [57–59]. In one of the first phase 2 dose-finding clinical trials [60], SHPT patients under hemodialysis treatment were randomized by Bell and colleagues to one of three different study treatment regimens: etelcalcetide 5 mg or placebo for 2 weeks (cohort 1); etelcalcetide 10 mg or placebo for 4 weeks (cohort 2); and etelcalcetide 5 mg or placebo for 4 weeks (cohort 3) [60]. Mean percent change of PTH from baseline was statistically significant (p < 0.05) and dose-dependent [60], reaching − 49% and + 29% in subjects treated with etelcalcetide 10 mg and placebo, respectively, in cohort 2, and − 33% and + 2% for etelcalcetide 5 mg and placebo, respectively, in cohort 3 [60]. The proportions of patients who achieved a 30% reduction in PTH levels from baseline were 76% in the etelcalcetide 10 mg group versus 10% in the placebo group (p < 0.0001). The proportion was lower in the etelcalcetide 5 mg group, equaling 54% versus 15% in the placebo group [60]. Finally, the numbers of patients achieving PTH levels below 300 pg/ml at the end of the study were 67% in the etelcalcetide 10 mg group and 46% in the etelcalcetide 5 mg group [60].

A second study conducted in Japan confirmed these findings even expanding them, as it suggested that the dose-dependent reduction of PTH levels might be associated with a corresponding effect on markers of bone neo-formation (bone-specific alkaline phosphatase) and bone resorption (tartrate-resistant acid phosphatase 5b) [61]. In the two phase 3 studies versus placebo [59], etelcalcetide was given three times weekly to 508 patients with moderate to severe SHPT as compared to 515 patients who were given placebo. At the end of the 26-week study duration, active treatment significantly reduced the levels of PTH (75% versus 9% in the placebo group) and of FGF-23 (70% versus 30% in the placebo group), and it also improved other bone mineral metabolism markers [59]. A post hoc analysis of the placebo-controlled study [62] evaluated the efficacy of etelcalcetide in achieving levels of PTH below 300 pg/ml according to specific baseline PTH levels. The results show that 69.2% of patients with baseline PTH < 600 pg/ml succeeded in achieving levels of PTH ≤ 300 pg/ml in the efficacy evaluation period; such proportion is reduced to as low as 48,9% and 29,5% for patients with baseline PTH from 600 to 1000 pg/ml and > 1000 pg/ml, respectively. These data are resulting from a symmetrical decrease of PTH levels compared to baseline by 54.2%, 58.2% and 55.5% in the three categories of patients with baseline PTH < 600 pg/ml, from 600 to 1000 pg/ml, and > 1000 pg/ml, respectively [62].

The phase 3 study recently published by Block and colleagues [58] showed that etelcalcetide was both non-inferior and superior to cinacalcet in controlling serum levels of PTH. A total of 683 patients from dialysis centers in Europe and the US were enrolled in this double-blind, double-dummy study, of whom 340 were treated with intravenous etelcalcetide and 343 with oral cinacalcet [58]. At the end of the 26-week follow-up period, etelcalcetide was shown to be non-inferior to cinacalcet with regard to the proportion of subjects whose PTH levels were reduced at least by 30% from baseline (68.2% vs. 57.7% of patients treated with etelcalcetide and cinacalcet, respectively, non-inferiority p value < 0.001) [58] and superior to cinacalcet with regard to the proportion of patients whose PTH levels were reduced at least by 50% (52.4% vs. 40.2% of patients treated with etelcalcetide and cinacalcet, respectively, p = 0.001) [58]. It has to be noted that all patient subgroups in which the efficacy of etelcalcetide was tested have shown the same effect of the new drug on PTH control [58]. A summary of key results from phase 3 studies is shown in Table 6.

**Table 6:** Summary of key results from phase 3 studies on etelcalcetide

Etelcalcetide
69% of patients with PTH < 600 pg/ml reached target < 300 pg/ml
Etelcalcetide reduces PTH, calcium and phosphate
Etelcalcetide showed superiority vs. cinacalcet with regard to the proportion of patients with > 30% and > 50% reduction of mean PTH from baseline during the EAP
Decreased serum calcium was mild to moderate, transient and rarely caused the discontinuation of the drug (Parsabiv^®^ European Public Assessment Report. EMA/664198/2016. September 2016)

The safety profile of etelcalcetide appears to be comparable to the one reported for cinacalcet [57–60]. Adverse events reported during the treatment with etelcalcetide in clinical trials seem to be for the most part correlated to the mechanism of action of calcimimetic drugs. The most important risk for etelcalcetide is the induction of hypocalcemia or events that might occur as a consequence of a decrease in serum calcium (for example, prolongation of QTc interval). The rate of these adverse events, in particular hypocalcemia or decrease of serum calcium to levels that are not considered as true hypocalcemia, is slightly higher than with cinacalcet. This is likely due to the higher potency of the new drug (Table 7).

**Table 7:** Incidence (%) of the most common drug-related adverse events

More frequent adverse event	Clinical trials versus placebo [59]		Clinical trials versus cinacalcet [58]	
	Placebo (N = 513)	Etelcalcetide (N = 503)	Cinacalcet (N = 341)	Etelcalcetide (N = 338)
Diarrhea	8.6	10.7	10.3	6.2
Nausea	6.2	10.7	22.6	18.3
Vomiting	5.1	8.9	13.8	13.3
Decreased serum calcium	10.1	63.8	59.8	68.9
Hypocalcemia^a^	0.2	7.0	2.3	5.0
Hypokalemia	3.1	4.4	5.3	3.8
Muscle spasms	6.6	11.5	5.9	6.5
Paresthesia	1.2	6.2	2.6	3.3
Arterial hypotension	–	–	2.9	6.8

^a^Hypocalcemia defined as serum calcium levels adjusted for albumin < 8.3 mg/dl

During phase 2 and phase 3 clinical trials, a total of 1655 subjects received at least one dose of etelcalcetide, and 499 of these received etelcalcetide for more than 1 year [55, 57–59, 63]. In placebo-controlled trials, the most common adverse event was asymptomatic hypocalcemia (occurring in 63.8% of patients treated with etelcalcetide vs. 10.1% of patients treated with placebo). Based on Kaplan–Meier estimates, the median time to the onset of the first event of hypocalcemia is around 9.6 weeks [55, 57–59, 63]. The tolerability profile of etelcalcetide was basically comparable to that of cinacalcet. The most commonly reported adverse events were asymptomatic hypocalcemia and gastrointestinal disorders [58, 59]. In particular, asymptomatic hypocalcemia was reported in 68.9% of patients treated with etelcalcetide and in 59.8% of those who were treated with cinacalcet. In both trials, hypocalcemia was controlled by administration of larger doses of calcium salts or vitamin D supplements and/or etelcalcetide dose reduction or interruption. While there is not consensus, it is plausible that lower levels of serum calcium in absence of symptoms can be tolerated. On the other hand, calcimimetic rather than calcium or vitamin D dose adjustment may be the best course of action in case of symptomatic hypocalcemia [35]. Gastrointestinal events (diarrhea, nausea and vomiting) were reported in 46.7% of patients treated with cinacalcet and 37.8% of patients treated with etelcalcetide (Table 6) [58, 59]. Although intravenous administration bypasses the gastrointestinal tract, etelcalcetide only partially reduced gastrointestinal adverse events (nausea and diarrhea) (Table 6) observed with the use of cinacalcet. These effects are likely due to the systemic activation of CaSR, rather than at the level of the gastrointestinal mucosa [64]. Nevertheless, these symptoms are generally mild in severity and improve with etelcalcetide dose reduction or drug interruption.

In placebo-controlled clinical trials, prolongation of QT interval (500 ms) secondary to hypocalcemia was observed in 4.8% of patients treated with etelcalcetide and 1.9% of subjects on placebo. Close monitoring of calcium levels is thus needed in patients with risk factors like congenital long QT syndrome, previous history of QT prolongation, familial anamnesis of long QT syndrome, sudden cardiac death and other medical conditions (data from European Medicines Agency (EMA): Parsabiv summary of product characteristics. Available from: http://www.ema.europa.eu, accessed November 30, 2016) or dugs (metoclopramide, antibiotics, etc.) that can predispose to QT interval prolongation. Also, caution should be exerted in the context of potential drug-to-drug interactions that can displace etelcalcetide from albumin binding and potentially worsen hypocalcemia or hypoglycemia when etelcalcetide is administered in diabetic patients with SHPT.

In dialysis patients, who undergo dialysis sessions three times weekly, regular monitoring of electrolytes and CKD-MBD biochemical markers seems sufficient to prevent the occurrence of severe adverse events. In this regard, the open label one-year extension of the three previously described phase 3 studies further corroborates the risk-to-benefit profile of etelcalcetide, without raising any additional safety concerns associated with longer-term exposure to the drug [62].

Etelcalcetide was associated with greater-than-placebo reductions in bone mineral metabolism markers (bone alkaline phosphatase and type 1 collagen C-terminal telopeptide) and fibroblast growth factor 23 (exploratory endpoints) at the end of the study (week 27) in placebo-controlled registration trials as well [59].

## Closing remarks

Pharmacological treatment of SHPT has made progress in the last years. The introduction of targeted therapies like VDR and CaSR selective modulators is offering more chances for an appropriate control of serum PTH levels, in particular in patients with CKD undergoing dialysis treatment. Emerging intravenous therapies for SHPT like etelcalcetide (AMG 416) might improve patients’ therapeutic compliance, reduce the number of drugs to take orally at home so as to simplify dosing schedules, and increase the likelihood to reach treatment goals suggested by the guidelines for the management of CKD-MBD (Table 2).

The question remains unanswered whether emerging SHPT treatments will prove they can reduce the risk of cardiovascular morbidity and mortality in CKD-G5D. Emerging therapies seem effective in reducing PTH, restoring mineral homeostasis, improving therapeutic compliance, and they might help create prerequisite conditions to improve long-term outcomes in dialysis patients with SHPT. However, the final answer to such a question can only come from randomized clinical trials on the new drugs, like etelcalcetide.
